# Draft Genome Sequence of Vibrio chagasii 18LP, Isolated from Gilthead Seabream (Sparus aurata) Larvae Reared in Aquaculture

**DOI:** 10.1128/MRA.00658-21

**Published:** 2021-09-16

**Authors:** Gracinda M. M. Sanches-Fernandes, Gianmaria Califano, Tina Keller-Costa, Sara Castanho, Florbela Soares, Laura Ribeiro, Pedro Pousão-Ferreira, Leonardo Mata, Rodrigo Costa

**Affiliations:** a Institute for Bioengineering and Biosciences (iBB), Instituto Superior Técnico (IST), Universidade de Lisboa, Lisbon, Portugal; b Associate Laboratory i4HB—Institute for Health and Bioeconomy at Instituto Superior Técnico, Universidade de Lisboa, Lisbon, Portugal; c Centre of Marine Sciences (CCMAR), University of Algarve, Faro, Portugal; d Institute for Inorganic and Analytical Chemistry, Friedrich-Schiller-Universität Jena, Jena, Germany; e Portuguese Institute for the Ocean and Atmosphere, Aquaculture Research Station, Olhão, Portugal; f Lawrence Berkeley National Laboratory, Department of Energy-Joint Genome Institute (DOE-JGI), UC Berkeley, Berkeley, California, USA; Loyola University Chicago

## Abstract

We report the draft genome sequence of Vibrio chagasii strain 18LP, isolated from gilthead seabream larvae at a fish hatchery research station in Portugal. The genome presents numerous features underlying opportunistic behavior, including genes coding for toxin biosynthesis and tolerance, host cell invasion, and heavy metal resistance.

## ANNOUNCEMENT

Vibrio spp. are ubiquitous in the marine environment and may cause disease outbreaks, leading to high economic losses in aquaculture (1, 2). Vibrio chagasii, for instance, has been isolated from seawater, sediments, sea bass, turbot larvae, and rotifers and *Artemia* spp. (3, 4), the latter usually provided as live feed to fish larvae in hatchery stations. V. chagasii has recently been identified as a causal agent of disease in marine invertebrates such as oysters and mussels (5–8).

To improve our understanding of the virulence factors within the species, we report the genome sequence of V. chagasii 18LP. Counts of *Vibrio* colonies in gilthead sea bream larvae (34 days after hatching) were estimated by spread-plating of larva-derived homogenates onto thiosulfate-citrate-bile salts-sucrose agar (TCBS; Oxoid, USA) after 7 days of incubation at 22°C (9). Strain 18LP was thereafter isolated and identified by 16S rRNA gene sequencing as described elsewhere (10). For genome sequencing, the Wizard genomic DNA purification kit (Promega, USA) was used to extract DNA from a pure culture grown in marine broth for 2 days at 19°C (10).

A genome library was constructed using the Illumina Nextera XT DNA library preparation kit (insert length, 450 bp). Paired-end sequencing was conducted on the Illumina HiSeq 2500 platform at BaseClear (The Netherlands). Default parameters were applied for all bioinformatics tools used, unless stated otherwise. FASTQ sequence files were generated using the Illumina Casava v1.8.3 pipeline. Reads containing adapters were removed using an in-house filtering protocol. The sequencing output was 466.3 Mb. The read quality was enhanced by trimming the low-quality bases using the “Trim sequences” option in CLC Genomics Workbench v7.0.4. The sequence reads were assembled into 49 contigs with the “*de novo* assembly” option within CLC Genomics Workbench v7.0.4. The optimal k-mer size was automatically determined using KmerGenie v1.6213 (11). Scaffolding of the preassembled contigs was performed using the SSPACE Premium v2.3 scaffolder (12). Automated partial closure of gaps within the scaffolds was performed using GapFiller v1.10 (13). V. chagasii 18LP possesses 95.5% average nucleotide identity with the type strain V. chagasii LMG 21353 according to the Microbial Genomes Atlas database (14). Genome annotation was performed using the Rapid Annotation using Subsystem Technology (RAST) v2.0 server, under the RASTtk scheme (15). Table 1 summarizes the general features of the V. chagasii 18LP genome.

**TABLE 1:** General features of the Vibrio chagasii 18LP genome

Feature	Description or statistic
Strain	V. chagasii 18LP
Host species	Sparus aurata
No. of reads	3,700,846
Read length (bp)	126
Genome size (Mb)	5.41
GC content (%)	44.4
Genome coverage (×)	86.2
No. of contigs	49
Contig *N*_50_ (bp)	350,607
Completeness (%)	95.5
Contamination (%)	1.8
No. of coding sequences	4,897
No. of RNAs	76
GenBank accession no.	GCF_903995485.1
SRA accession no.	ERR6053174

V. chagasii 18LP possesses 59 coding sequences (CDSs) assigned to the virulence, disease, and defense subsystem within RAST. Of these, 5 CDSs involved in cholera toxin biosynthesis and regulation were annotated. With regard to resistance to antibiotics, bacteriocins, and toxic compounds, strain 18LP possesses genes encoding colicin E2 tolerance (1 CDS), synthesis of multidrug resistance efflux pumps (6 CDSs) and beta-lactamase (1 CDS), and resistance to fluoroquinolones (2 CDSs). Moreover, 24 genes encoding copper homeostasis and tolerance were found, along with cobalt-zinc-cadmium (5 CDSs) and chromium (1 CDS) resistance genes. Finally, 14 CDSs required for active host invasion and intracellular resistance, involved in protein synthesis (small-subunit [SSU] and large-subunit [LSU] ribosomal proteins), DNA transcription, and quinolinate biosynthesis, were found in the genome of V. chagasii 18LP.

### Data availability.

This genome sequence was deposited at the European Nucleotide Archive (ENA) under the BioProject accession number PRJEB9149, BioSample accession number SAMEA7110813, RefSeq assembly accession number GCF_903995485.1, and SRA run accession number ERR6053174. The annotation reported in this study is available on the RAST platform for guest users under the job number 877705 and ID 6666666.587972.
